# Systematic improvements in lentiviral transduction of primary human natural killer cells undergoing *ex vivo* expansion

**DOI:** 10.1016/j.omtm.2021.01.008

**Published:** 2021-01-20

**Authors:** David S.J. Allan, Mala Chakraborty, Giacomo C. Waller, Michael J. Hochman, Akkapon Poolcharoen, Robert N. Reger, Richard W. Childs

**Affiliations:** 1 Laboratory of Transplantation Immunotherapy, Cellular and Molecular Therapeutics Branch, National Heart Lung and Blood Institute, National Institutes of Health, Bethesda, MD 20892, USA

## Abstract

Transduction of primary human natural killer (NK) cells with lentiviral vectors has historically been challenging. We sought to evaluate multiple parameters to optimize lentiviral transduction of human peripheral blood NK cells being expanded to large numbers using a good manufacturing practice (GMP)-compliant protocol that utilizes irradiated lymphoblastoid (LCL) feeder cells. Although prestimulation of NK cells with interleukin (IL)-2 for 2 or more days facilitated transduction with vesicular stomatitis virus glycoprotein (VSVG)-pseudotyped lentivirus, there was a subsequent impairment in the capacity of transduced NK cells to proliferate when stimulated with LCL feeder cells. In contrast, incubation of human NK cells with LCL feeder cells plus IL-2 before transduction in the presence of the TBK1 inhibitor BX795 resulted in efficient lentiviral integration (mean of 23% transgene^+^ NK cells) and successful subsequent proliferation of the transduced cells. Investigation of multiple internal promoter sequences within the same lentiviral vector revealed differences in percentage and level of transgene expression per NK cell. Bicistronic lentiviral vectors encoding both GFP and proteins suitable for the isolation of transduced cells with magnetic beads led to efficient transgene expression in NK cells. The optimized approaches described herein provide a template for protocols that generate large numbers of fully functional and highly purified lentivirus-transduced NK cells for clinical trials.

## Graphical Abstract

**Figure:**
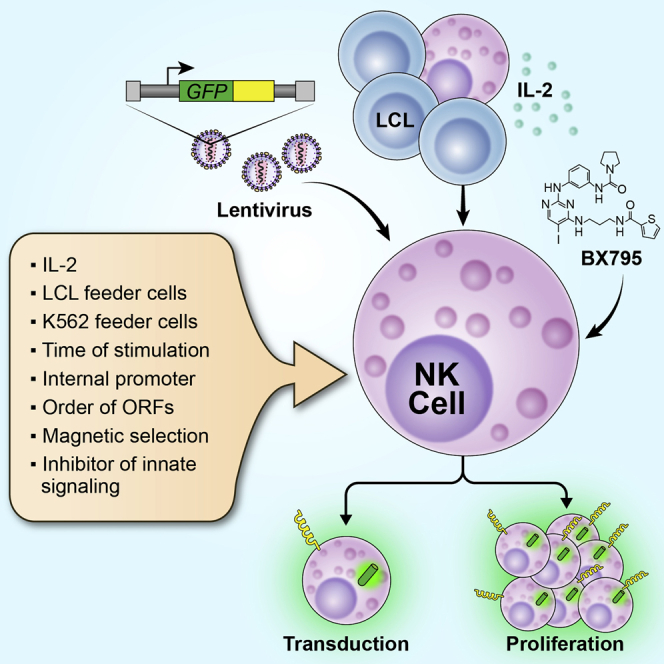

## Teaser

By systematically evaluating multiple potential influencing factors, Childs and colleagues merge protocols to optimize lentiviral transduction and *ex vivo* expansion of human NK cells via co-culture with irradiated LCL feeder cells to produce large numbers of genetically modified NK cells for cancer immunotherapy.

## Introduction

It has been recognized since the 1970s that natural killer (NK) cells show unprimed cytotoxicity toward hematological malignant cells.^1^^,^^2^ Because of their distinct mechanism of tumor recognition, depending on the balance between ligation of activating and inhibitory cell surface receptors and low risk of initiating graft-versus-host responses, NK cells have unique potential for immunotherapeutic intervention in cancer. Miller et al.^3^ demonstrated that infusion of interleukin (IL)-2-activated haploidentical NK cells, after lymphodepleting chemotherapy, resulted in complete remissions in some acute myeloid leukemia (AML) patients. The response rate of this therapy was improved by depletion of regulatory T cells.^4^ Other studies have shown clinical efficacy of treatments with allogeneic NK cells activated with IL-2^5^ or stimulated with IL-15, IL-12, and IL-18^6^ in AML or myelodysplastic syndrome (MDS) patients. We have examined the clinical effects of *ex vivo*-expanded autologous NK cells in concert with treatment with the proteasome inhibitor bortezomib (ClinicalTrials.gov: NCT00720785),^7^ which has been shown to sensitize tumors to NK cell cytotoxicity.^8^^,^^9^ NK cells also contribute to the efficacy observed with monoclonal biologics such as rituximab, as genetic variants of the NK cell receptor CD16, which mediates antibody-dependent cytotoxicity, correlate with anti-tumor responses.^10^^,^^11^

Therapeutic NK cells may benefit from genetic modification to express genes that improve their function in the tumor microenvironment, trafficking to tumors, or persistence after adoptive transfer.^12^ NK cells may also be re-targeted to recognize different tumor antigens via expression of chimeric antigen receptors (CARs).^13^^,^^14^ Studies in these areas have been hampered by the relative difficulty of viral modification of NK cells in comparison with T cells. mRNA transfection of NK cells is an efficient alternative^15^^,^^16^ but only induces transient expression of the transferred gene. Significant strides have been made in improving modification of NK cells with gamma retroviral vectors.^13^^,^^14^^,^^17^, ^18^, ^19^, ^20^ Transduction with gamma retroviral vectors depends on cell cycling, so protocols have incorporated conditions to induce NK cell proliferation such as co-culture with activated T cells^17^ or irradiated K562 target cells modified with cell surface-expressed cytokines and the 4-1BBL costimulatory molecule.^13^^,^^19^^,^^20^ Similar methods have facilitated CAR expression in NK cells,^13^^,^^14^^,^^19^ allowing their translation to the clinic. In one recent trial, 7 of 11 chronic lymphocytic leukemia (CLL) and lymphoma patients receiving NK cells, modified to express anti-CD19 CAR and IL-15, achieved complete remission without evidence of cytokine release syndrome.^21^

In comparison with gamma retroviruses, lentiviral vectors show certain advantages, such as reduced potential for genotoxic insertional mutagenesis.^22^ However, transduction of NK cells with lentiviral vectors has proven challenging. Since 1999, it has been recognized that high-dose IL-2 treatment of NK cells could facilitate some transduction of NK cells with lentivirus pseudotyped with vesicular stomatitis virus glycoprotein (VSVG).^23^, ^24^, ^25^, ^26^ Exposure to combinations of cytokines, such as IL-2 plus IL-12^25^^,^^26^ or IL-2 with IL-21,^27^ were shown to further augment lentiviral transduction of mouse and human NK cells. In addition, Sutlu et al.^27^ innovatively demonstrated that altering of the cellular anti-viral response, by treating NK cells with an inhibitor of innate immune signaling pathways, also potentiated lentiviral insertion into NK cells. Despite these advances, the proportion of NK cells successfully modified with lentiviral vectors has lagged behind the results achieved with other cell types or with other viruses.

A clinically compatible protocol to expand human NK cell numbers by approximately 500-fold via incubation with an irradiated lymphoblastoid (LCL) cell line and IL-2 has been developed previously by our group^28^ and used in the aforementioned clinical trial together with bortezomib (ClinicalTrials.gov: NCT00720785).^7^ In this study, we set out to systematically evaluate different parameters to increase the transduction of human NK cells with lentiviral vectors and to merge the transduction and expansion protocols to achieve a method that efficiently produces large numbers of genetically modified NK cells.

## Results

### IL-2 stimulation enhances lentiviral transduction of primary human NK cells

**Figure 1:**
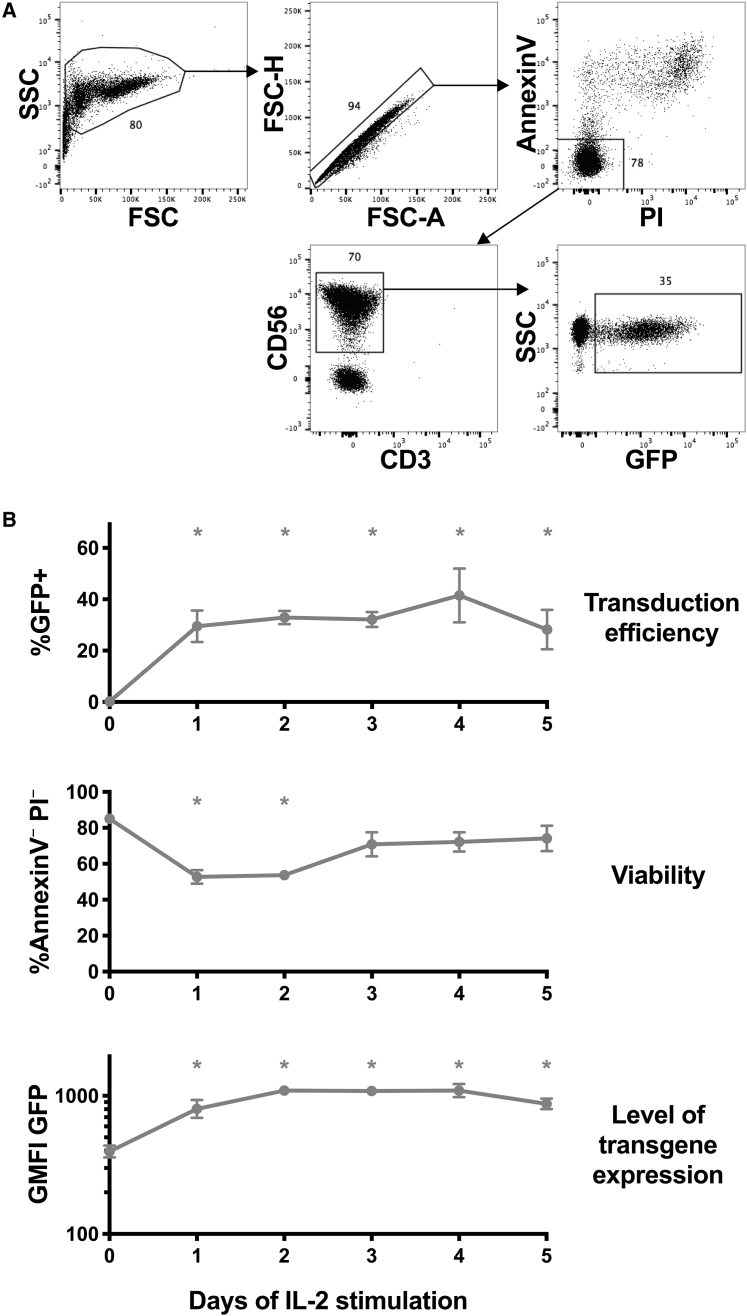
Stimulation with IL-2 improves lentiviral transduction of primary human NK cells Enriched NK cells from peripheral blood mononuclear cells (PBMC) were cultured with 500 U/mL IL-2 for 0–5 days as indicated, before transduction with pLV-PGK-GFP vector at a 20:1 MOI in RetroNectin-coated plates. 3 days after transduction, in each case, cells were assayed for GFP expression and viability by flow cytometry. (A) Typical gating of GFP^+^ NK cells. (B) Mean and SEM (antilog of mean and SEM of log_10_-transformed values for geometric mean fluorescence intensity [GMFI] GFP) of experiments from three donors are shown. ∗p < 0.05 compared to cells without IL-2 stimulation (day 0) by Dunnett’s multiple comparisons test after one-way ANOVA (of log_10_-transformed values for GMFI). The percentage of annexin V^–^propidium iodide (PI)^–^ cells depicted in (B) was tabulated on gated CD56^+^CD3^–^ cells.

Multiple factors were examined for their potential to facilitate transduction of primary blood NK cells with lentiviral vectors. The effect of prestimulation with IL-2 for different time periods was investigated. NK cells were isolated from human buffy coat samples and incubated with 500 U/mL human IL-2 in the same culture medium previously used in our protocol for clinical NK cell preparation.^28^ NK cells were transduced with VSVG-pseudotyped pLV-PGK-GFP, a lentiviral vector encoding mouse phosphoglycerate kinase (PGK) internal promoter and enhanced GFP as a reporter of integration. In these experiments, all periods of IL-2 prestimulation (1–5 days) resulted in significant increases in GFP^+^ NK cells (mean of 33%) compared to NK cells transduced immediately after isolation (mean of 0.2%), evaluated 3 days after transduction in each case (Figures 1A and 1B). The viability of NK cells was depressed somewhat for cells that had been stimulated with IL-2 for only 1–2 days before transduction as assessed by annexin V and propidium iodide flow cytometry staining 3 days after transduction. However, NK cells stimulated for 3 or more days before viral transduction retained high viability (Figure 1B). Importantly, this combination of IL-2 prestimulation for 2–3 days followed by lentiviral transduction resulted in transduction efficiencies in the 26%–61% range (mean of 35%, n = 12 from six donors). Similar transductions were evaluated in the presence of polybrene, protamine, and retronectin reagents known to increase viral association with cells; NK cells transduced with retronectin trended toward a higher percentage of GFP^+^ cells with good viability after culture, although differences were not statistically significant (Figure S1).

### Lentiviral vectors containing different internal promoters show varying levels of stable transgene expression in human primary NK cells

**Figure 2:**
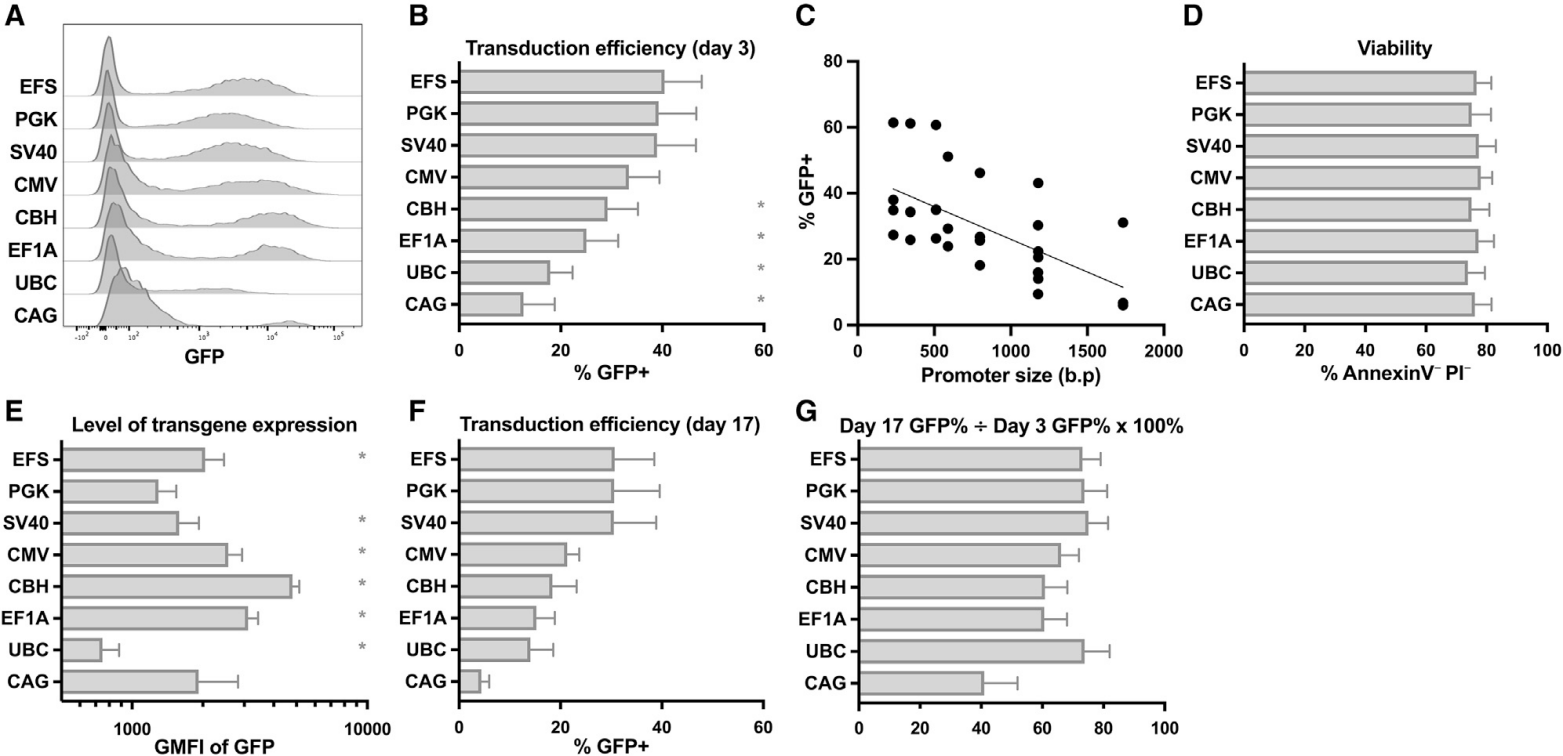
Lentiviral vectors containing different promoter sequences show variable levels of transduction and transgene expression in human NK cells NK cells enriched from PBMC were stimulated with IL-2 for 3 days before transduction with lentiviral vectors containing the indicated promoter sequences and GFP. (A–E) 3 days after transduction, NK cells were examined by flow cytometry for GFP expression (percent and GMFI) (A, B, C, and E) and viability by PI/annexin V staining (D). (C) GFP% is plotted compared to promoter size in base pairs (R^2^ = 0.40, p < 0.0001 by linear regression). (F) Irradiated LCL feeder cells were added to the transduced NK cells and co-cultured for an additional 14 days (17 days post-transduction), at which time GFP% was evaluated again. (G) Stability of transgene expression in NK cells over time was quantified by dividing the GFP% at day 17 post-transduction by the GFP% at day 3 post-transduction and expressed as a percent. Mean and SEM (antilog of mean and SEM of log_10_-transformed values for GMFI) are shown in each case for experiments from four donors. ∗p < 0.05 compared to the previously used PGK promoter containing vector by Dunnett’s multiple comparisons test after one-way ANOVA (of log_10_-transformed values for GMFI). See also Table S1.

Hypothesizing that the inefficiencies of lentiviral expression previously observed in NK cells could be caused by silencing of integrated sequences, we evaluated multiple internal promoter sequences, in the context of the same lentiviral vector backbone, for their capacity to drive stable gene expression in NK cells. Each viral preparation was titrated on HeLa cells to achieve comparable infectivity and was used at a 20:1 multiplicity of infection (MOI). All lentiviral vectors with different internal promoters were able to transduce human NK cells; however, those containing the PGK promoter, the short form of the EF1A promoter (EFS), the SV40 viral promoter, or the human cytomegalovirus (CMV) immediate early promoter showed the highest percentages of GFP^+^ NK cells (Figures 2A and 2B). Promoter size was directly related to the length of the viral genome and correlated with the percentage of GFP positivity (Figure 2C; Table S1) but did not have an impact on cellular viability (Figure 2D). Constructs containing different internal promoters resulted in different levels of GFP transgene expression in transduced NK cells, with the short form chicken β-actin with the CMV enhancer (CBH) promoter, the EF1A promoter, the CMV promoter, and the EFS promoter vectors all showing higher GFP geometric mean florescence intensity (GMFI) compared to the PGK promoter vector (Figures 2A and 2E). Transduced cells were cultured with LCL feeder cells and IL-2 and maintained for several weeks. Most constructs showed fairly stable expression of GFP (Figure 2F) as measured by the ratio of GFP percentage at day 17 post-transduction divided by the GFP percentage at day 3 post-transduction (Figure 2G), arguing against the occurrence of widespread silencing of integrated viral DNA sequences. EFS promoter constructs were chosen for further study due to the combination of a high percentage of GFP^+^ cells and high GFP protein level per cell, as well as achieving stable transgene expression over time.

### Order of genes within lentiviral vectors affects transduction of human NK cells

**Figure 3:**
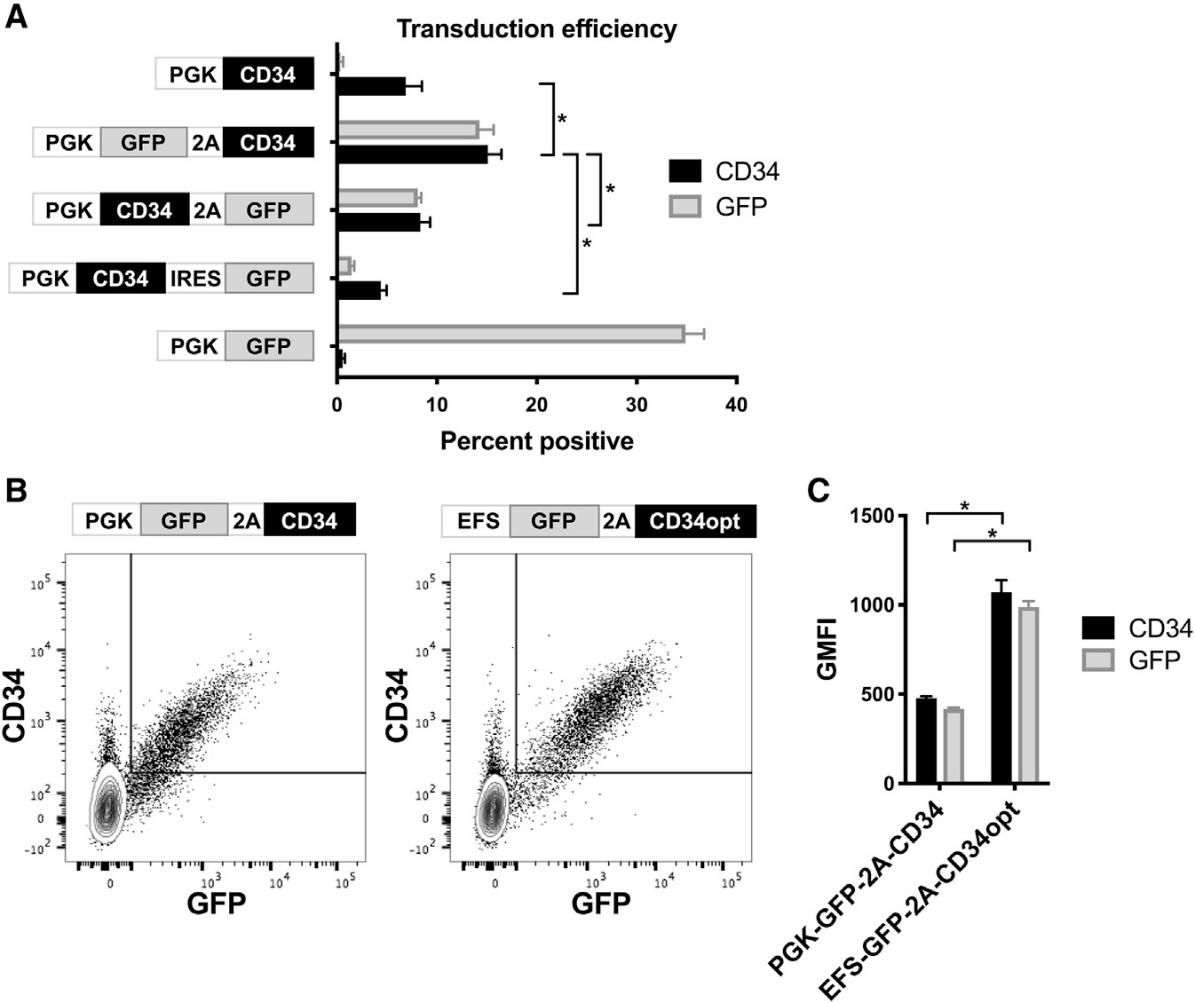
Arrangement of insert sequences within the lentiviral vector affects transduction efficiency in human NK cells NK cells from PBMC were stimulated with IL-2 for 3 days before transduction with lentiviral vectors containing the indicated insert sequences encoding combinations of PGK promoter, GFP, P2A-linker, IRES, truncated CD34 protein, and codon-optimized truncated CD34 protein (CD34opt). After 3 additional days, NK cells were measured by flow cytometry for GFP and surface expression of CD34. (A) Mean and SEM of experiments from three donors are shown. (B) Typical staining comparing constructs containing different internal promoters and original versus codon-optimized truncated CD34. (C) Means and SEM of gated GFP^+^ cells from experiments from three donors as in (B). ∗p < 0.05 for selected comparisons by Tukey’s multiple comparisons test after one-way ANOVA (A) or paired t test (C). Also see Figure S2.

Similar vectors and conditions were evaluated for their ability to facilitate expression of genes other than GFP in primary NK cells. Bicistronic vectors were created to express genes of interest along with a selectable reporter protein, such as truncated human CD34.^29^ In several constructs, GFP and truncated CD34 were arranged in different orders, linked by the P2A autocatalytic cleavage sequence or internal ribosome entry site (IRES) (Figure 3A; Figure S2). Interestingly, transduction of human NK cells with constructs containing the CD34 sequence individually showed much lower percentages of CD34^+^ cells compared to GFP^+^ percentages observed when transducing with a vector encoding GFP alone (Figure 3A). However, inclusion of the CD34 sequence after the GFP sequence (linked by P2A) in the viral vector resulted in the generation of a considerably higher percentage of CD34^+^ NK cells (Figure 3A). Vectors with the opposite order (linked by P2A or IRES) resulted in fewer CD34^+^ and GFP^+^ cells (Figure 3A). These effects on NK cells appeared disproportionate to HeLa cells, which were used to titrate all vectors to achieve equivalent transduction levels. Vectors encoding genes of interest distal to GFP were examined in further detail, as they appeared to be more effective in transducing NK cells (Figure 3A and data not shown). Figures 3B and 3C show additional improvement of the bicistronic vector by exchanging EFS for the PGK promoter (see data in Figure 2) and including a codon-optimized sequence for truncated CD34; transduction of human NK cells with this vector resulted in higher expression of GFP and CD34 on a per cell basis (Figures 3B and 3C).

### Integration of lentiviral transduction into an NK cell expansion protocol: effects of prestimulation with an LCL cell line before transduction

**Figure 4:**
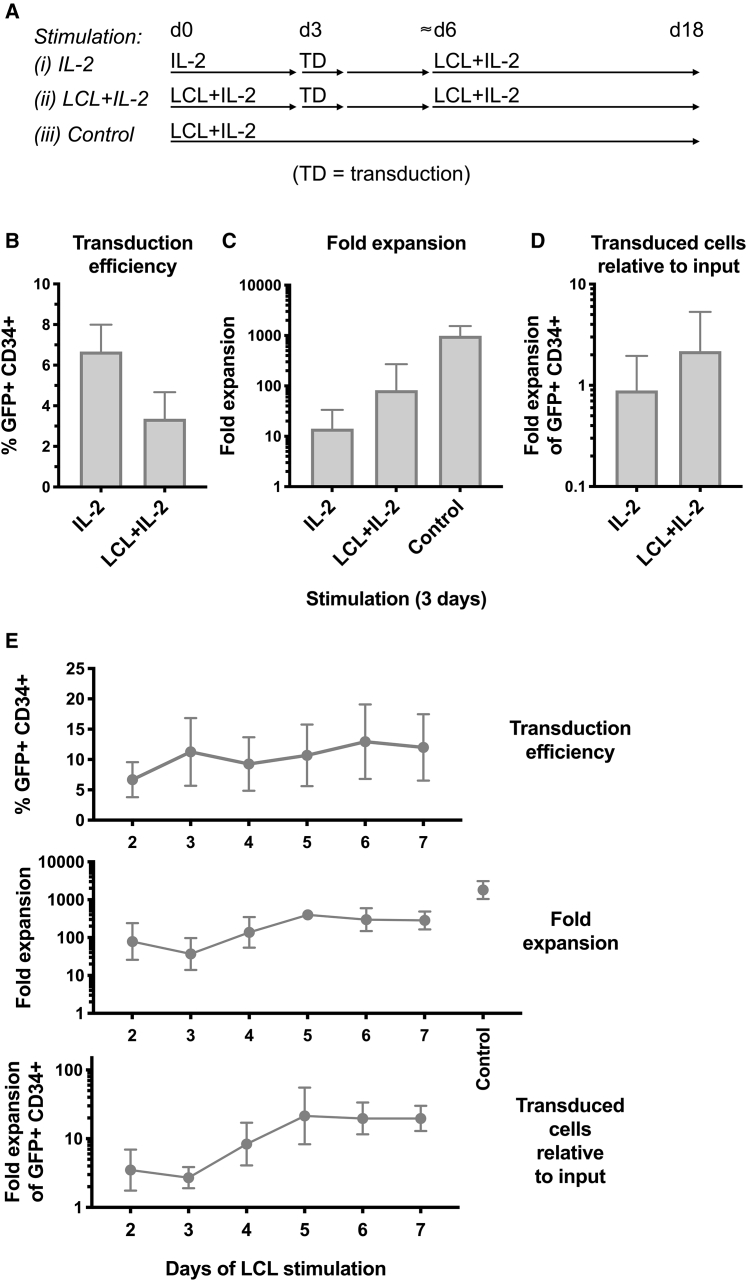
Effects of prestimulation of human NK cells with LCL feeder cells before lentiviral transduction on overall yield of transduced cells after culture (A–D) NK cells from PBMC were stimulated with (i) IL-2 or (ii) IL-2+LCL feeder cells for 3 days. NK cells were removed from co-culture with anti-CD56 beads before transduction with pLV-EFS-GFP-2A-CD34 viral particles at a 20:1 MOI. After 2–4 additional days, irradiated LCL feeders were added to both conditions (i and ii), and NK cells were cultured until day 18–19 from the start of the experiment. (B) NK cells were examined by flow cytometry for GFP and CD34 transgene expression. Mean and SEM are shown for experiments from five donors. (C) NK cell numbers were counted frequently throughout culture, and the fold expansion was calculated. Antilog of mean and SEM of log_10_-transformed values are shown in comparison with control expansions of un-transduced NK cells from the same donors, which received LCL on day 0 only. (D) Overall yield of transduced cells relative to input was calculated by multiplying the fold expansion of all cells with the GFP^+^CD34^+^ fraction for each condition. Antilog of mean and SEM of log_10_-transformed values are shown. (E) Similar experiments were performed to determine the optimal period of stimulation with LCL cells before transduction (pLV-EFS-GFP-2A-CD34). Transduced NK cells were stimulated with irradiated LCL feeders a second time in each case 3 days after transduction, and cultures were followed until day 21 from the start of the experiment. The percentage of transduced cells, fold expansion of cells, and overall yield of transduced cells were determined as in (B)–(D) for experiments from three donors.

As shown above, prestimulation with IL-2 facilitated transduction of primary NK cells (Figure 1). Since our aim is the production of large numbers of genetically modified NK cells for immunotherapeutic use, the subsequent capacity of these transduced NK cells to proliferate was investigated. After 3 days of IL-2 stimulation and transduction, and then another approximately 3 days to allow viral integration, NK cells were subjected to our published NK cell-expansion protocol^28^ by co-culturing with IL-2 and irradiated LCL feeder cells (at an LCL/NK ratio of 10:1) (see (i) IL-2 diagram in Figure 4A). This was contrasted with an alternate procedure, wherein NK cells were prestimulated with LCL feeder cells with IL-2 (rather than IL-2 alone) before transduction and similar culture thereafter (Figure 4A and discussed below). Although stimulation with IL-2 led to measurable transgene expression (Figure 4B), the overall increase in cell number of these transduced cultures was substantially less than those of untransduced control NK cells, which were initially co-cultured with LCL feeder cells without virus (Figures 4A and 4C). Following IL-2 stimulation and transduction, the ratio of resulting GFP^+^CD34^+^ transduced cells compared to the starting number of NK cells showed an unimpressive geometric average of 0.9 (measured 15 or 16 days after transduction) (Figure 4D). We hypothesized that the loss of efficient proliferation was more likely the consequence of using IL-2 alone for 3 days to stimulate NK cells before they were transduced and stimulated with irradiated LCL feeder cells, although lentiviral exposure appeared to cause an additional deficit (Figure S3).

Attempting to develop a strategy that could be used to overcome the observed deficiency in proliferation of transduced NK cells, we investigated a protocol that utilized LCL feeder cell stimulation before transduction. Primary human NK cells were co-cultured with IL-2 plus irradiated LCL cells. After 3 days, NK cells were isolated from cellular debris and any remaining LCL cells using anti-CD56 magnetic beads, followed by lentiviral transduction. About 3 days later, transduced NK cells were restimulated with irradiated LCL feeder cells and NK cell numbers were tracked (see (ii) LCL+IL-2 diagram in Figure 4A). This regimen, wherein NK cells experienced prestimulation with LCL cells, appeared to result in a lower percentage of transduced cells (Figure 4B) with a higher fold expansion of NK cell numbers (Figure 4C), which ultimately resulted in a trend toward an increased overall yield of GFP^+^CD34^+^ transduced cells (Figure 4D) compared with IL-2 alone prestimulation.

To investigate the optimal number of days of prestimulation with LCL feeder cells, primary human NK cells were co-cultured with IL-2 plus LCL cells for 2–7 days, before exposure to viral transduction. After 21 total days in culture, there was a trend toward the overall yield of GFP^+^CD34^+^ transduced cells being higher in cultures prestimulated for ≥5 days with LCL cells plus IL-2 before transduction (Figure 4E). In similar experiments where primary NK cells were prestimulated with LCL feeder cells and IL-2, we observed that magnetic bead separation of NK cells (from the remaining irradiated LCL cells and cellular debris) prior to lentiviral transduction did not increase the final yield of transduced NK cells (Figure S4).

### Treatment with TBK1 inhibitor BX795 after LCL cell stimulation results in the greatest yield of transduced human NK cells

**Figure 5:**
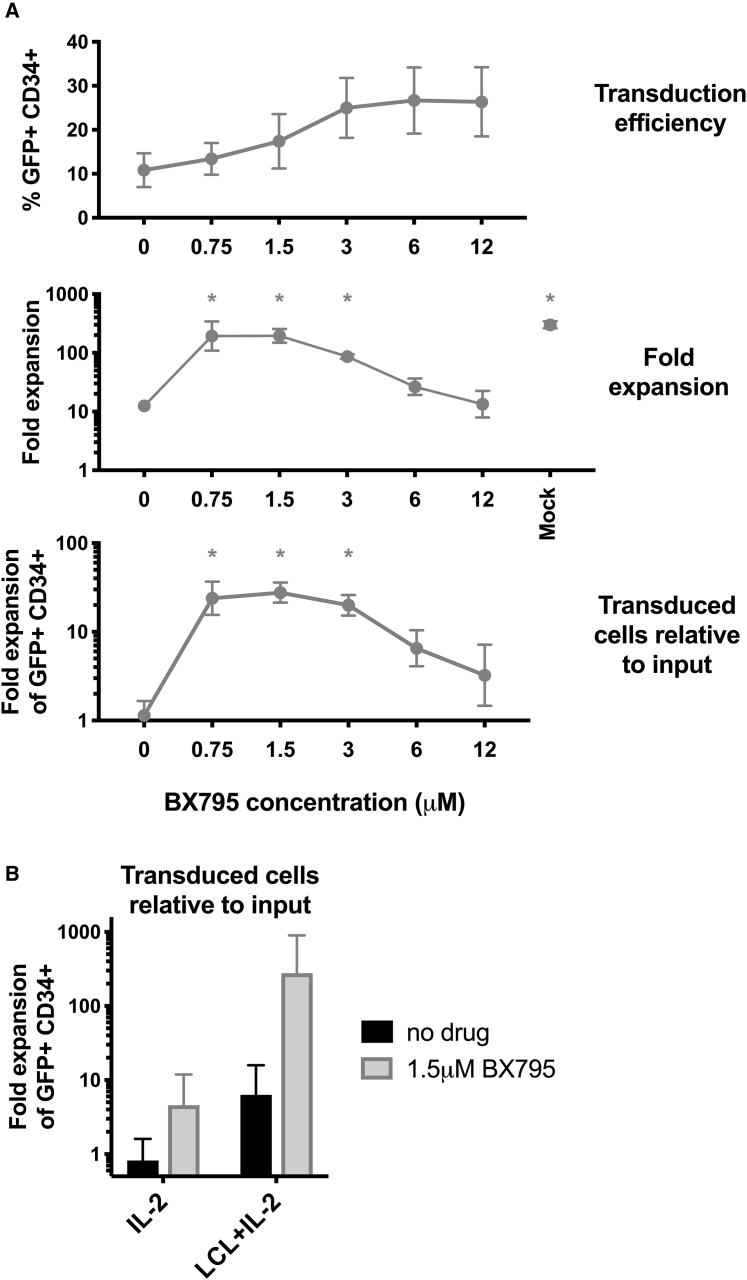
BX795 combined with prestimulation with LCL feeder cells increases subsequent proliferation of lentiviral-transduced, *ex vivo*-expanded human NK cells (A) NK cells from PBMC were stimulated with LCL cells plus IL-2 for 5 days. NK cells were removed from co-culture with anti-CD56 beads before transduction with pLV-EFS-GFP-2A-CD34 viral particles in the presence of varying concentrations of BX795 as indicated. After 1 day, NK cells were exchanged into medium without BX795. After 2-3 additional days, LCL cells were added again and NK cells cultured until day 21 from the start of experiment, with cell numbers monitored throughout. The same NK cells that underwent comparable manipulations (mock transduction) and culture without added virus and without BX795 are labeled as mock. NK cells were assayed for GFP and CD34 expression by flow cytometry. The yield of transduced cells relative to input cells was calculated by multiplying overall fold expansion of cells times the fraction of GFP^+^CD34^+^ cells. Mean and SEM are shown (antilog of mean and SEM of log_10_-transformed values for fold expansions) for experiments from three donors. ∗p < 0.05 compared to conditions lacking drug by Dunnett’s multiple comparisons test after one-way ANOVA of log_10_-transformed values. (B) Similar experiments were performed to compare cells stimulated for 4-5 days with (i) IL-2 or (ii) IL-2+LCL feeder cells before transduction with or without addition of 1.5 μM BX795. The overall yield of transduced cells relative to input cells was tabulated as above for four experiments from four blood donors (three using viral particles prepared with second-generation lentiviral packaging plasmids and one with third-generation packaging). Addition of LCL feeders, addition of 1.5 μM BX795, and their interaction all showed p < 0.05 in a two-way ANOVA (of log_10_-transformed values).

It has been demonstrated previously that treatment of NK cells with the BX795 inhibitor compound, which affects innate immune signaling downstream of pattern recognition receptors, can enhance productive lentiviral integration.^27^ This compound was tested in the context of our NK cell transduction/proliferation protocol. Human NK cells, prestimulated for 5 days with LCL cells and IL-2, were exposed to lentiviral particles in the presence of different concentrations of BX795. Compound and virus were washed away after 1 day, and the NK cells were then tracked for proliferation as described previously. Interestingly, BX795 concentrations ≥3 μM caused highest percentages of successfully integrated lentiviral vector (Figure 5A), but these BX795 concentrations led to sub-maximal NK cell proliferation (Figure 5A). Optimal yields of transduced cells appeared to be achieved at approximately 1.5 μM BX795 (Figure 5A). Supplementation with 1.5 μM BX795 augmented the proliferation of NK cells prestimulated with LCL plus IL-2 before viral exposure more than those prestimulated with IL-2 alone (Figure 5B). Figure 5B shows the cumulative improvements in the overall yield of transduced NK cells (compared to input cells) produced by adding LCL prestimulation supplemented with an optimal concentration of BX795.

An alternative K562 feeder cell line similar to that used for clinical-grade NK cell expansions^21^^,^^30^^,^^31^ (K562 cells expressing 4-1BBL and surface-bound IL-21) was also assessed in the context of this regimen and yielded comparable results to our experiments using LCL cells (Figure S5). These transductions were performed in retronectin-coated plates, but the alternate enhancer polybrene was also investigated using this protocol, showing equivalent or greater percentages of transduced NK cells, but concentration-dependent reductions in NK cell viability were observed (data not shown); proliferation of NK cells transduced with polybrene was not assessed. Transductions of NK cells with lentiviral particles prepared with either second-generation or third-generation packaging plasmids showed similar results (data not shown).

### Bicistronic lentiviral vectors achieve robust cell-surface protein expression in fully functional expanded human NK cells

**Figure 6:**
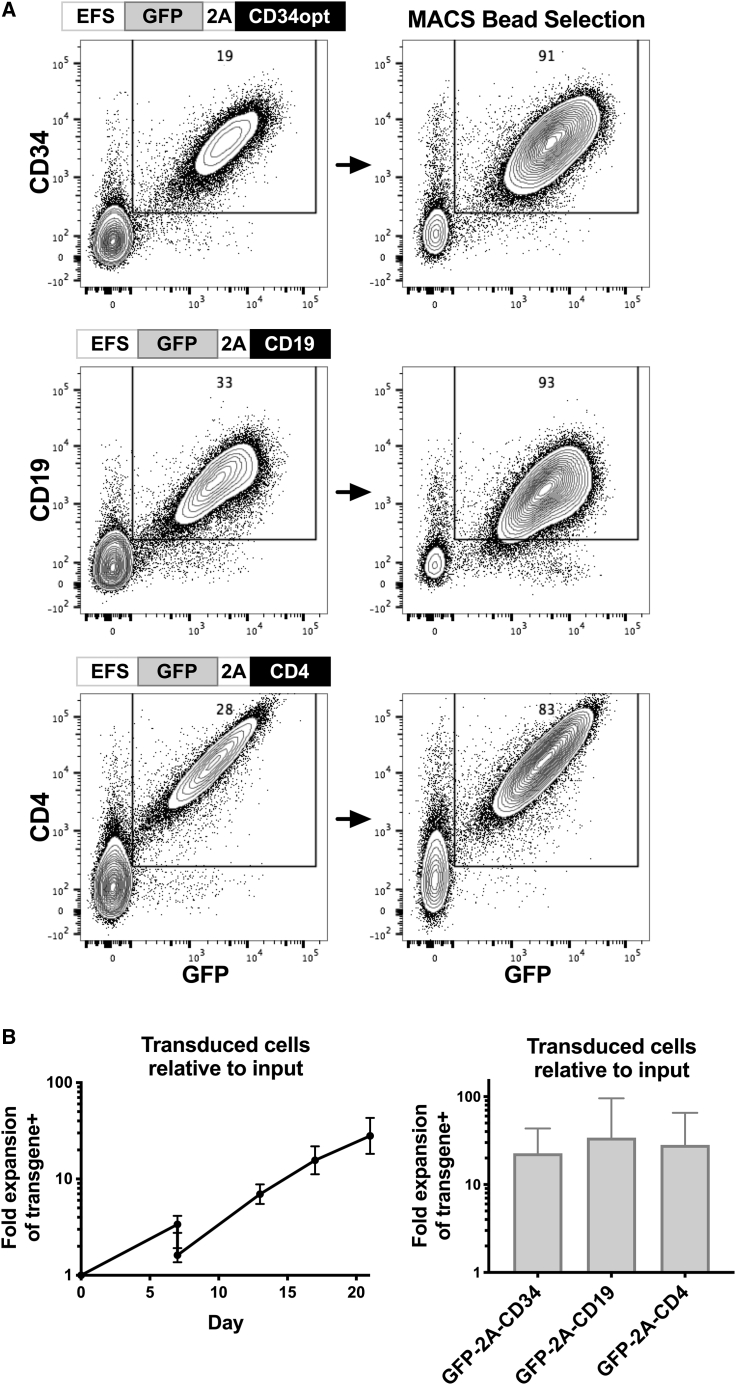
Optimizing an LCL protocol that facilitates lentiviral-transduced expression of surface proteins and subsequent proliferation of positively selected human NK cells Blood NK cells were stimulated with LCL plus IL-2 for 4–5 days, then transduced in the presence of 1.5 μM BX795 with lentiviral vectors encoding codon-optimized truncated CD34, truncated CD19, or truncated CD4 as indicated. After 1 day, cells were exchanged into medium without BX795. After 2 additional days, transduced NK cells were selected using MACS beads recognizing CD34, CD19, or CD4. Positively selected NK cells were cultured with LCL feeder cells, and the proliferation of cells was followed until 21 days from the start of the experiments. (A) Flow cytometry evaluation of GFP and transgene expression before and after MACS bead selection. (B) Cell numbers were monitored throughout, and the overall yield of transduced cells relative to input cells was calculated by multiplying the fold expansion of cells times the fraction positive for both GFP and transgene expression. Antilog of mean and SEM of log_10_-transformed values of experiments from three donors are shown. Results from one donor utilized pLV-EFS-GFP-2A-CD4 prepared with third-generation lentiviral packaging plasmids, while others used second-generation plasmids.

To determine whether these improved stimulation and transduction conditions could facilitate transduction of varied gene products in human NK cells, experiments were performed with vectors encoding truncated CD19,^32^ truncated CD4, as well as truncated CD34 (all distal to GFP sequences) (Figure 6A). In each case, significant levels of cell-surface expression of these proteins that were co-expressed with GFP were observed (Figure 6A). CD19^+^GFP^+^ NK cells averaged 33%, CD4^+^GFP^+^ cells 25%, and CD34^+^GFP^+^ cells averaged 23% (in experiments from three donors). Each of these molecules can be recognized by clinical-grade magnetic beads, which allow for the selection and purification of human NK cells that have been successfully transduced (Figure 6A). Importantly, these transduced NK cells retained the capacity to proliferate before and after bead-mediated selection (Figure 6B). At day 21, this protocol yielded GFP^+^protein^+^ transduced cells that expanded 10- to 371-fold (geometric average of 55, median of 54, n = 13) relative to the number of input NK cells in the absence of selection and 10- to 267-fold (geometric average of 40, median of 42, n = 13) when bead selection was incorporated into the protocol (five donors).

**Figure 7:**
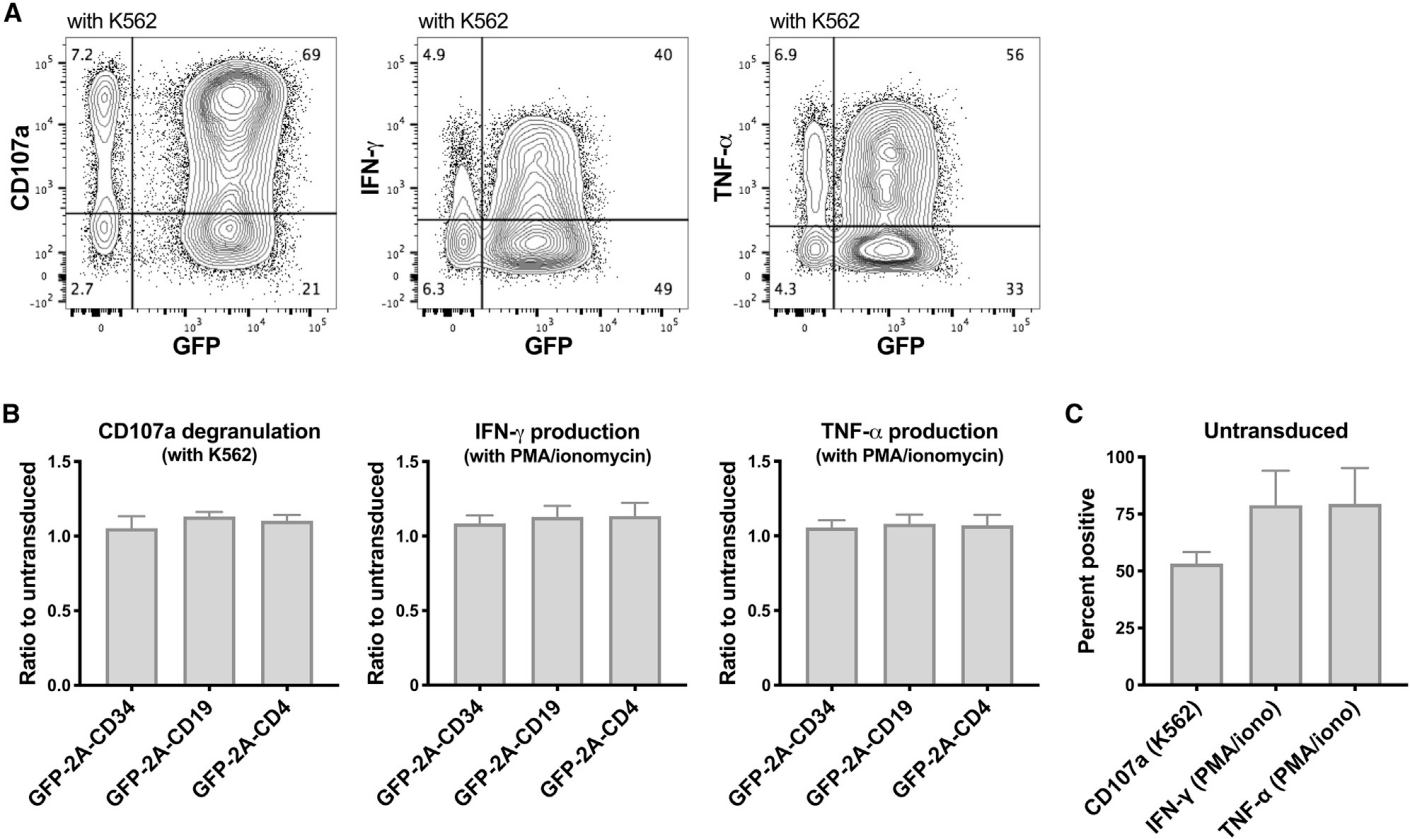
Lentiviral-transduced human NK cells expanded with LCL feeders maintain full degranulation plus IFN-γ and TNF-α production (A) Human NK cells transduced and expanded as described in Figure 6 were plated with K562 target cells (1:1 ratio) or treated with PMA plus ionomycin as indicated. Degranulation was assayed after 2 h with anti-CD107a antibody, and cytokine production was measured after 4 h by intracellular flow cytometry staining for IFN-γ and TNF-α. (B) Shown are mean and SEM of percentages normalized to values measured with control un-transduced NK cells from the same donors expanded with LCL feeder cells (C).

The functional capacity of NK cells genetically modified and expanded in cell number using this protocol was evaluated. The ratios of NK cell degranulation (measured by CD107a surface expression) and interferon (IFN)-γ and tumor necrosis factor (TNF)-α cytokine production upon exposure to K562 targets (or phorbol 12-myristate 13-acetate [PMA]/ionomycin exposure) averaged >100% when comparing transduced GFP^+^ cells with control NK cells that had been expanded with LCL cells and IL-2 without transduction in separate cultures from the same donors (Figure 7). Taken together, this optimized protocol results in efficient transduction while preserving the proliferative capacity of transduced cells, thus providing a clinically applicable strategy to produce large numbers of highly functional genetically modified effector NK cells.

## Discussion

To augment the immunotherapeutic potential of NK cells, we sought to develop a method that improves the lentiviral transduction of human NK cells being expanded to large numbers for clinical use. Multiple factors potentially affecting viral insertion were systematically investigated, including stimulation of NK cells with cytokines or feeder cell lines, optimal periods of stimulation, type of feeder cell, variety of enhancer, inclusion and concentration of an inhibitor of cell-intrinsic immune signalling, sequence of the internal promoter in the lentiviral construct, and order of inserted open reading frames. In each case, results from multiple donors were used to ultimately develop an optimized protocol, wherein NK cells were stimulated with irradiated LCL feeder cells and 500 U/mL IL-2 for 5 days, followed by lentiviral transduction in the presence of 1.5 μM BX795 and retronectin, followed by a restimulation with irradiated LCL feeder cells. This protocol resulted in an average of 23% of expanded NK cells showing expression of a bicistronic transgene, with NK proliferative capacity preserved to allow for the generation of transduced cells that expanded a median 54-fold compared to starting NK cell numbers.

These results provide several insights regarding the proliferative potential of NK cells undergoing viral modification. Incubation of human NK cells with IL-2 facilitated productive transduction by lentiviral vectors (Figure 1), as reported previously.^23^, ^24^, ^25^, ^26^, ^27^ However, after IL-2 prestimulation, NK cells that were subsequently transduced did not proliferate efficiently when subjected to conditions used in our expansion protocol utilizing irradiated LCL feeder cells plus IL-2 (Figure 4). Similarly, human NK cells pretreated with IL-2, but lacking viral exposure, showed a similar proliferative deficit upon subsequent culture with irradiated LCL feeders (Figure S3). This implies that exposure to IL-2 itself induces NK cells into a cellular state that precludes subsequent rapid proliferation through pathways utilized by Epstein-Barr virus (EBV)-transformed feeders to induce NK cell cycling. Remarkably, we found that prestimulation of human NK cells with irradiated LCL feeders plus IL-2 before transduction preserved their ability for subsequent proliferation *in vitro* following transduction compared with prestimulation with IL-2 only.

Inclusion of the compound BX795 during lentiviral exposure increased the fraction of human NK cells showing transgene expression (Figure 5), as has been previously reported.^27^ BX795 blocks the catalytic activity of TBK1/IKKε (IKBKE) kinases by impeding their phosphorylation and therefore may affect signaling downstream of several innate pattern recognition receptors.^33^ Remarkably, we observed that the concentrations of BX795 that most increased the percentage of transduced NK cells hindered subsequent NK cell proliferation to suboptimal levels (Figure 5), possibly due to a direct toxic effect of the drug or through other mechanisms not explored in our analysis that altered innate immune responses. Although the percentage of transduced cells was increased to a lesser degree when lower concentrations of BX795 were utilized, NK cell proliferative capacity after transduction was maintained, which had the net effect of optimizing the yield of transduced NK cells harvested from cultures (Figure 5). These responses to BX795 suggest that human NK cells may mount an intrinsic immune response to lentiviral infection that inhibits productive viral integration/expression and the subsequent proliferation of NK cells. In this context, virus may enter cells without integration or trigger responses without entry, potentially influencing cellular proliferation despite the absence of expression of any transgene. Reverse-transcribed lentiviral DNA has the potential to be recognized by cyclic guanosine monophosphate-adenosine monophosphate synthase (cGAS) and PQBP1,^34^^,^^35^ while lentiviral RNA may also be sensed by endosomal TLR3 or cytoplasmic MDA5 (IFIH1) or RIG-I (DDX58);^36^ all of these proteins show signaling through TBK-1, which can be influenced by BX795. A confounding factor may be that viral preparations, differing in relative content of infectious versus inactive viral particles, may differentially induce NK cell-intrinsic immune responses. Pattern recognition receptors and TBK-1 signaling could also be triggered by residual DNA plasmids and transduction reagents in viral preparations, suggesting that more highly purified viral preparations (e.g., chromatographic isolation) might show additional improvements. Such factors could potentially account for the variability in transduction efficiency observed in different studies when unstimulated human NK cells were transduced (Figure 1).^25^^,^^27^ These variables, along with altered transgene sequences and inter-donor differences, may explain the unequal magnitude of BX795-mediated effects between experiments (Figure 5B; Figure S5). From an evolutionarily standpoint, it would be advantageous for NK cells to possess finely tuned immune sensor mechanisms to detect and stop their own infection with retroviruses, since NK cells function as front-line sentinels responding directly to infected cells. In our culture system, using lower concentrations of BX795 provided the appropriate level of inhibition of cell-intrinsic responses to augment viral transduction while preserving the capacity of transduced NK cells to subsequently proliferate. Notably, our experiments showed that both (1) prestimulation with LCL feeder cells and IL-2 and (2) transduction in the presence of BX795 synergized to improve the yield of transduced NK cells (Figure 5).

Human NK cells showed varied percentages of transduction when comparing lentiviral constructs differing in internal promoter sequences (Figure 2) or with different orders of transgenes in the vector (Figure 3). Since these constructs differ in specific nucleotide sequences, it is interesting to speculate that sequence changes may influence detection by pattern recognition receptors, thereby influencing cell-intrinsic innate responses that could block viral integration or expression. Alternatively, the percentage of transgene^+^ NK cells correlated with the size of the inserted promoter sequence, pointing to viral RNA size as a factor impacting transduction of NK cells (Figure 2C). These effects were selectively observed in NK cells compared with HeLa tumor cells, since viral preparations were titrated to approximately equal infectivity on HeLa cells. Whether these reflect specific differences between NK cells and HeLa cells or between NK cells and other cell types remains to be investigated. Viral constructs containing transgenes encoded distal to GFP appeared to be more efficient at transducing human NK cells (Figure 3), which could reflect particular features of the enhanced GFP sequence that make it efficiently translated or continually transcribed without locus silencing. Unfortunately, strong and rapid anti-GFP immune responses have been observed in non-human primates when GFP-expressing cells were adoptively transferred without myeloablative conditioning regimens.^37^^,^^38^ As such, these GFP^+^ vectors may work well in preclinical models but would ultimately be replaced by vectors lacking GFP for clinical use. Recent publications have indicated that lentiviral particles produced with the envelope protein of baboon endogenous retrovirus are more efficient at transducing NK cells compared with those produced with the VSVG envelope.^39^^,^^40^ It will be interesting to determine whether lentiviral particles pseudotyped with alternative envelope glycoproteins will further enhance the yield of transduced NK cells using our methods. Constructs derived from another genus of viruses, gamma retroviral vectors, when pseudotyped with feline endogenous virus RD114 or gibbon ape leukemia virus envelope have shown very promising results.^13^^,^^14^^,^^17^, ^18^, ^19^, ^20^, ^21^

The sequential systematic examination of multiple factors that may affect human NK cell transduction has resulted in a distilled procedure that achieves clinically applicable percentages of NK cells expressing lentiviral-encoded proteins with the proliferative capacity of transduced NK cells being preserved. The resulting optimized protocol described herein, which generates fully functional transduced NK cells, may serve as a platform for genetic modification of human NK cells to improve their anti-cancer function in clinical trials. The ability to reproducibly transduce human NK cells will also have great utility as a tool in basic scientific studies of NK cell biology.

## Materials and methods

### Cell lines

The EBV-transformed B cell line SMI-LCL was described previously.^41^ K562 cells expressing 4-1BBL and IL-21 (clone 46) were kindly provided by Dr. Katayoun Rezvani (MD Anderson Cancer Center, Houston, TX, USA). Both were cultured in RPMI 1640 medium supplemented with 10% heat-inactivated fetal bovine serum (FBS).

### Isolation and culture of primary human NK cells

De-identified human peripheral blood buffy coats were provided by the Department of Transfusion Medicine, NIH Clinical Center. The NIH Office of Human Subjects Research Protections determined that use of this material was excepted from the requirements of institutional review board (IRB) review. Primary NK cells were isolated using RosetteSep human NK cell enrichment cocktail (STEMCELL Technologies, BC, Canada) with lymphocyte separation medium (MP Biomedicals, Irvine, CA, USA) or by sequential CD3 depletion and CD56 selection using magnetic-activated cell sorting (MACS) beads (Miltenyi Biotec, Bergisch Gladbach, Germany). NK cells were cultured in X-VIVO 20 medium (Lonza, Basel, Switzerland) supplemented with 10% heat-inactivated human AB serum (Millipore-Sigma, MA, USA), 2 mM GlutaMAX (Thermo Fisher Scientific, Waltham, MA, USA), and 500 IU/ml IL-2 (teceleukin, Roche, Basel, Switzerland), refreshing at least half the medium every 1–5 days (usually every 2–3 days). LCL cells or K562 4-1BBL IL-21 cells were irradiated (100 Gy) and added to culture as indicated. Cell numbers were enumerated by trypan blue staining and microscopic counting or using a Luna-FL fluorescence cell counter (Logos Biosystems, Anyang, South Korea) with acridine orange and propidium iodide stains. Statistical comparisons of proliferation were assessed using GraphPad Prism 8.4.1 comparing log_10_(fold change) values.

### Production of lentiviral particles

Table S1 shows all lentiviral vectors used in this study, which were cloned according to our design by VectorBuilder (Chicago, IL, USA). Plasmid DNA was purified using a ZymoPURE II-EndoZero plasmid maxiprep kit (Zymo Research, Irvine, CA, USA). Unless otherwise specified, HEK293T cells in 125 cm^2^ flasks were transfected with Lipofectamine 3000 (Thermo Fisher Scientific) and DNA of lentiviral vector, second-generation packaging plasmid pCMV-dR8.2dvpr, and pCMV-VSVG at a 5:4:1 mass ratio. Where indicated, DNA of lentiviral vector, third-generation packaging plasmids pMDLg/pRRE and pRSV-Rev, plus pCMV-VSVG were used at a 5:2.5:1.25:1 mass ratio. dR8.2dvpr and pCMV-VSVG were gifts from Bob Weinberg via Addgene (Watertown, MA, USA), while pMDLg/pRRE and pRSV-Rev were gifts from Didier Trono via Addgene. Medium was exchanged after 6 h to Opti-MEM (Thermo Fisher Scientific) with 2 mM GlutaMAX and 5% FBS. Supernatants were collected 1 and 2 days later, then concentrated by precipitation with Lenti-X reagent (Takara Bio, Kusatsu, Japan) or by centrifugation at 18,600 × *g* relative centrifugal force (RCF) for 2 h at 4°C. Viral particles were resuspended in PBS or in X-VIVO 20 at 1/100 of the volume of the total supernatant. Viral titers were determined by serial dilution and transduction of HeLa cells in the presence of 8 μg/mL polybrene, and then flow cytometry for GFP expression 3 days later. Purification by Lenti-X produced higher titers (with HeLa cells) compared with ultracentrifugation. However, when matched to an equivalent MOI using the HeLa titration results, ultracentrifuged viral preparations resulted in greater percentages of transduced NK cells (data not shown).

### Lentiviral transduction of NK cells

NK cells were pretreated with IL-2 with or without feeder cells before transduction as indicated. Transduction of IL-2-stimulated NK cells using an MOI of 5 was found to result in 0.80-fold lower percentage of transduced NK cells on average compared with an MOI of 20, while an MOI of 100 resulted in 1.33-fold higher percentage (n = 16 from two blood donors using the varied lentiviral constructs from Figure 2) (data not shown). However, given the marginal benefit, subsequent experiments evaluating other variables impacting transduction were conducted using an MOI of 20 (unless otherwise indicated) to economically utilize viral stocks. Upon optimization, the protocol entailed the following: primary NK cells were stimulated with irradiated (100 Gy) SMI-LCL feeder cells at a 10:1 LCL/NK ratio with 500 U/mL IL-2 for 4–5 days. Non-tissue culture-treated plates were coated with RetroNectin (3.5–5 μg/cm^2^) (Takara Bio), blocked with 2% BSA for 30 min, washed with PBS, and then incubated with viral particles in NK cell culture medium (described above). The plate was then centrifuged at 2,000 × *g* for 1 h at 32°C and subsequently incubated 1 h at 37°C. NK cells were added (with 1.5 μM BX795 reagent [InvivoGen, San Diego, CA, USA] where indicated) and centrifuged at 1,000 × *g* for 10 min at 32°C (5 × 10^5^ or 2 × 10^6^ NK cells/well in 24- or 6-well plates, respectively). Culture medium was replaced the next day. After 1–3 days, LCL feeder cells were added at a 10:1 ratio to restimulate the NK cells. In some experiments, transduced NK cells were positively selected using anti-CD34, anti-CD4, or anti-CD19 MACS magnetic beads and LS columns (Miltenyi Biotec) before restimulation with LCL.

### Flow cytometry

NK cells were stained with antibodies recognizing CD56 (NCAM16.2), CD3 (UCHT1), CD34 (581), CD4 (RPA-T4), CD19 (HIB19), TNF-α (Mab 11), and IFN-γ (B27) from BD Biosciences (Franklin Lakes, NJ, USA), CD107a (H4A3), CD34 (561), and CXCR4 (12G5) from BioLegend (San Diego, CA, USA), along with propidium iodide (BD Biosciences), annexin V (BioLegend), and/or a Live/Dead fixable aqua dead cell stain kit (Thermo Fisher Scientific). Cells were examined on a BD Biosciences LSRFortessa instrument, and data were analyzed using FlowJo v10.1 (BD Biosciences). Statistical analyses were performed with GraphPad Prism 8.4.1.

### Degranulation and cytokine production assays

NK cells were stimulated by co-culture with K562 target cells at a 1:1 ratio. CD107a degranulation was assessed at 2 h by surface staining for flow cytometry. In parallel cultures, cytokine production was measured after 4 h (with GolgiStop and GolgiPlug [BD Biosciences] added at recommended concentrations within the first hour) by fixation with Cytofix/Cytoperm reagent (BD Biosciences) and intracellular staining for cytokine production.

## Supplemental information

Document S1. Figures S1–S5 and Table S1

Document S2. Article plus supplemental information
