# Supplementary material for: Systematic improvements in lentiviral transduction of primary human natural killer cells undergoing ex vivo expansion
Source: Mol Ther Methods Clin Dev. 2021 Jan 20;20:559–71. doi: 10.1016/j.omtm.2021.01.008 (PMC7890427; doi:10.1016/j.omtm.2021.01.008)
Supplement: Document S1. Figures S1–S5 and Table S1 [file mmc1.pdf]

OMTM, Volume *20*

Supplemental Information

Systematic improvements in lentiviral transduction of primary human natural killer cells undergoing *ex vivo* expansion

David S.J. Allan, Mala Chakraborty, Giacomo C. Waller, Michael J. Hochman, Akkapon Poolcharoen, Robert N. Reger, and Richard W. Childs

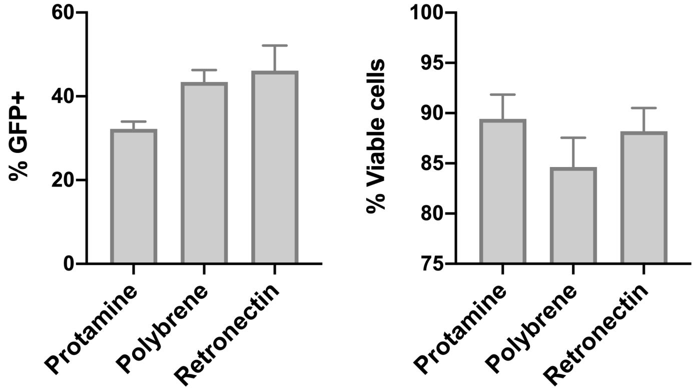

Figure S1. Effects of enhancers on transduction of primary human NK cells with lentiviral vectors. NK cells from PBMC were stimulated with 500 U/mL IL-2 (or IL-2 and 10-50 ng/mL IL-15) for 3-4 days then transduced with pLV-PGK-GFP in the presence of either protamine sulfate (10 μg/mL), polybrene (8 μg/mL), or using retronectin-coated plates known to enhance retroviral transduction. Several days later, NK cells were queried for the percentage of GFP^+ cells and viable cells by flow cytometry. Mean and SEM of experiments from 5 donors are shown. One-way ANOVA p values were > 0.05.

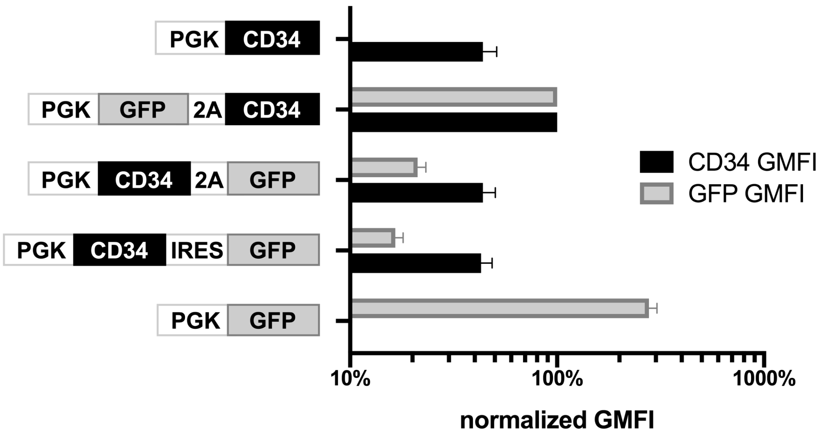

Figure S2. Arrangement of insert sequences within the lentiviral vector affects transduction efficiency in human NK cells. Further data from experiment depicted in Figure 3A. Geometric mean fluorescent intensity of gated $CD34^+$ or GFP^+ populations are shown, normalized in each experiment to the result obtained with pLV-PGK-GFP-2A-CD34 vector. Antilog of mean and SEM of $\log_{10}$ transformed values are shown.

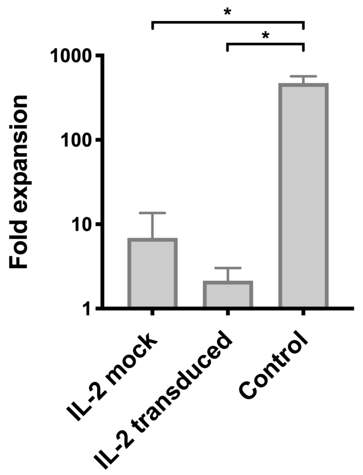

Figure S3. Primary human NK cells pre-stimulated with IL-2 lose the ability to proliferate efficiently upon subsequent exposure to irradiated LCL feeder cells. NK cells from PBMC were stimulated with IL-2 for 3-5 days, then subjected to (i) mock transduction without virus or (ii) transduction with pLV-EFS-GFP-2A-CD34 viral particles. After 2-3 additional days, irradiated LCL feeder cells were added to both conditions (i and ii). Control cultures of NK cells which received irradiated LCL feeders at the start of culture are shown for comparison. Overall proliferation of NK cells at day 19 of culture is depicted (antilog of mean and SEM of $\log_{10}$ transformed values of 3 experiments from 2 blood donors). * $p<0.05$ by Tukey's multiple comparisons test after one-way ANOVA of $\log_{10}$ transformed values.

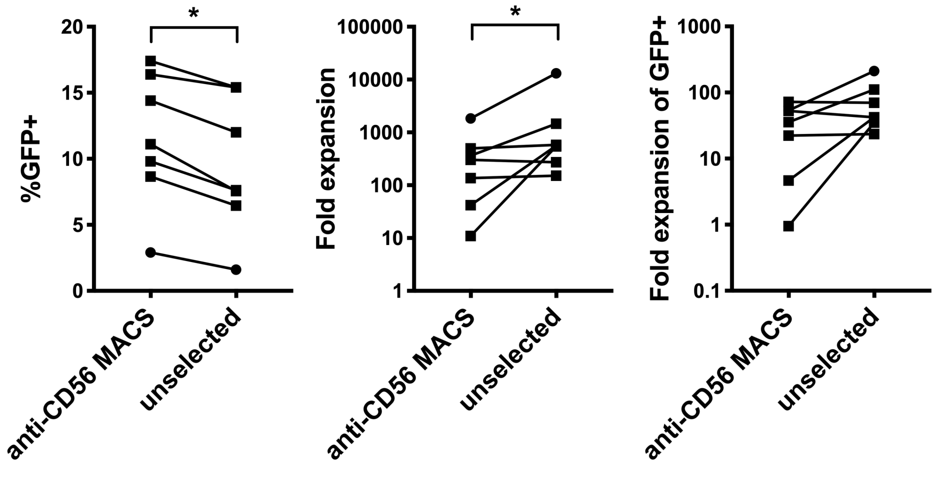

Figure S4. Magnetic bead separation of NK cells from irradiated LCLs before transduction does not improve yields of transduced NK cells. Peripheral blood NK cells were stimulated with irradiated LCL and IL-2 for 2-7 days, then selected with anti-CD56 MACS beads to isolate NK cells from remaining LCL and debris, or left unselected. Cells were then transduced with pLV-EFS-GFP-2A-CD34 (squares) or pLV-EFS-GFP-2A-CD4 (circles) viral particles. Three days later, LCL feeders were again added and NK cells cultured until day 21 from the start of the experiment. Percentages of cells expressing GFP were determined by flow cytometry. Fold expansion of total NK cell numbers or GFP^{+} NK cell numbers from the start of culture are shown. Results of 7 independent experiments from 2 blood donors are depicted. * $p<0.05$ by paired T test (of $\log_{10}$ transformed values for fold expansions).

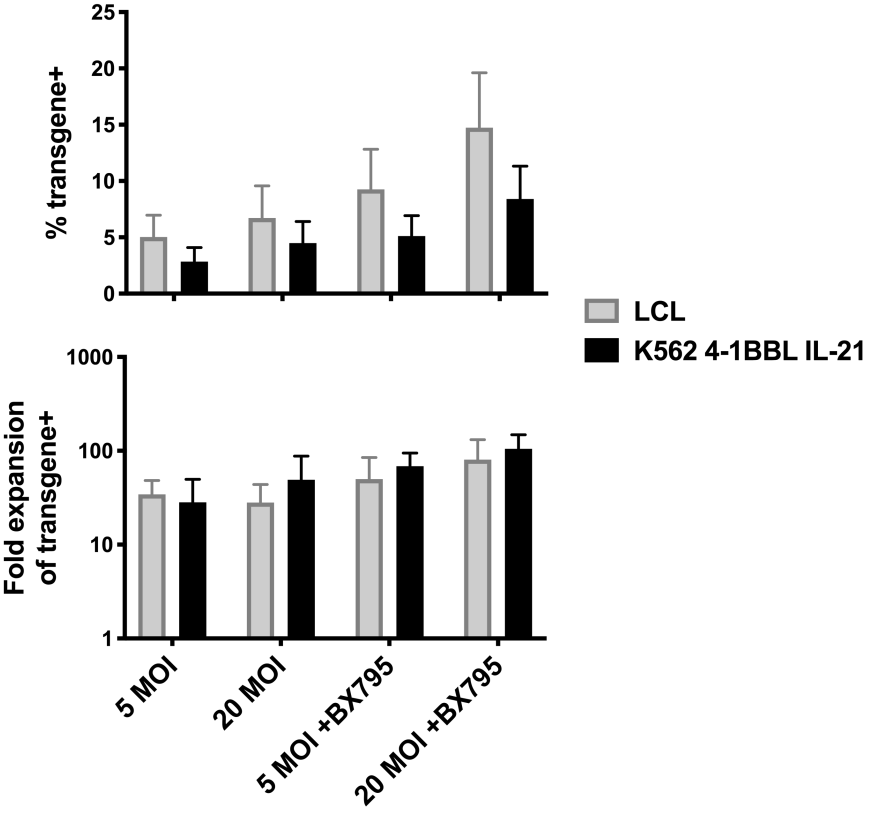

Figure S5. Effects of feeder cell type on NK cell transduction. Peripheral blood NK cells were stimulated with IL-2 and either LCL (10:1 ratio) or K562 4-1BBL IL-21 (2:1 ratio). After 4-5 days, cells were transduced with pLV-CD34-2A-CXCR4 at the indicated MOI with or without 1.5 μM BX795. 2-3 days later, feeder cells were added again. Mean and SEM percentages of cells expressing the CD34 transgene were determined by flow cytometry. Fold expansion of transgene^{+} (CD34^{+}) NK cell numbers from the start of culture to day 14 are shown (antilog of mean and SEM of $\log_{10}$ transformed values). Results from 3 blood donors are depicted. In a three-way ANOVA of $\log_{10}$(fold expansion of transgene^{+} cells) both the feeder variety and presence of BX795 were $p<0.05$. No factor was significant in an ANOVA of transgene%. pLV-CD34-2A-CXCR4 viral particles were generated with 3rd generation lentiviral packaging plasmids.

Table S1. Lentiviral vectors

Name	Vector Backbone	Insert	Internal promoter	Promoter size (b.p.)	Viral genome size
pLV-PGK-GFP	pLV[Exp]	EGFP	Mouse phosphoglycerate kinase 1 (PGK)	511	3942
pLV-CMV-GFP	pLV[Exp]	EGFP	Human cytomegalovirus (CMV) immediate/early	589	4020
pLV-EF1A-GFP	pLV[Exp]	EGFP	Human eukaryotic translation elongation factor 1α1 (EF1A)	1179	4610
pLV-EFS-GFP	pLV[Exp]	EGFP	Human EF1A short form (EFS)	232	3663
pLV-CAG-GFP	pLV[Exp]	EGFP	CMV early enhancer fused to modified chicken β-actin promoter (CAG)	1733	5164
pLV-CBh-GFP	pLV[Exp]	EGFP	CMV early enhancer fused to modified chicken β-actin promoter short form (CBh)	798	4229
pLV-SV40-GFP	pLV[Exp]	EGFP	Simian virus 40 enhancer/early (SV40)	344	3775
pLV-UBC-GFP	pLV[Exp]	EGFP	Human ubiquitin C (UBC)	1178	4609
pLV-PGK-CD34	pLV[Exp]	truncated human CD34 (tCD34)	PGK	511	4173
pLV-PGK-CD34-IRES-GFP	pLV[Exp]	tCD34; IRES; EGFP	PGK	511	5487
pLV-PGK-CD34-2A-GFP	pLV[Exp]	tCD34; P2A linker; EGFP	PGK	511	4956
pLV-PGK-GFP-2A-CD34	pLV[Exp]	EGFP; P2A; tCD34	PGK	511	4956
pLV-EFS-GFP-2A-CD34	pLV[Exp]	EGFP; P2A; codon optimized tCD34	EFS	232	4677
pLV-EFS-GFP-2A-CD4	pLV[Exp]	EGFP; P2A; truncated human CD4	EFS	232	5001
pLV-EFS-GFP-2A-CD19	pLV[Exp]	EGFP; P2A; truncated human CD19	EFS	232	4728
pLV-EFS-CD34-2A-CXCR4	pLV[Exp]	tCD34; P2A; truncated human CXCR4	EFS	232	4959
